# Inter- and intramolecular excimer circularly polarised luminescence of planar chiral paracyclophane-pyrene luminophores†

**DOI:** 10.1039/d0ra01552k

**Published:** 2020-03-20

**Authors:** Nobuyuki Hara, Motohiro Shizuma, Takunori Harada, Yoshitane Imai

**Affiliations:** Department of Applied Chemistry, Faculty of Science and Engineering, Kindai University 3-4-1 Kowakae, Higashi-Osaka Osaka 577-8502 Japan y-imai@apch.kindai.ac.jp; Department of Biochemistry, Osaka Research Institute of Industrial Science and Technology 1-6-50 Morinomiya, Joto-ku Osaka 536-8553 Japan; Department of Integrated Science and Technology, Faculty of Science and Technology, Oita University Dannoharu, 700 Oita City 870-1192 Japan

## Abstract

Two types of planar chiral [2,2]paracyclophane-pyrene luminophores (1 and 2) with different binding positions of the fluorescent pyrene units were synthesised. (*R*)/(*S*)-1 with 1-pyrene units exhibited green intermolecular excimer circularly polarised luminescence (CPL) at 530 nm in the KBr-pellet, but exhibited no CPL signal in dilute CHCl_3_ solution. In contrast, (*R*)/(*S*)-2 with 2-pyrene units exhibited a blue intramolecular excimer CPL at 450 nm in CHCl_3_ solution. This is the first example of using the binding position of pyrene and the external environment to tune the type (inter- or intramolecular) and chiroptical sign of excimer CPL.

## Abstract 2

Two types of planar chiral [2,2]paracyclophane-pyrene luminophores (1 and 2) with different binding positions of the fluorescent pyrene units were synthesised.

## Graphical abstract

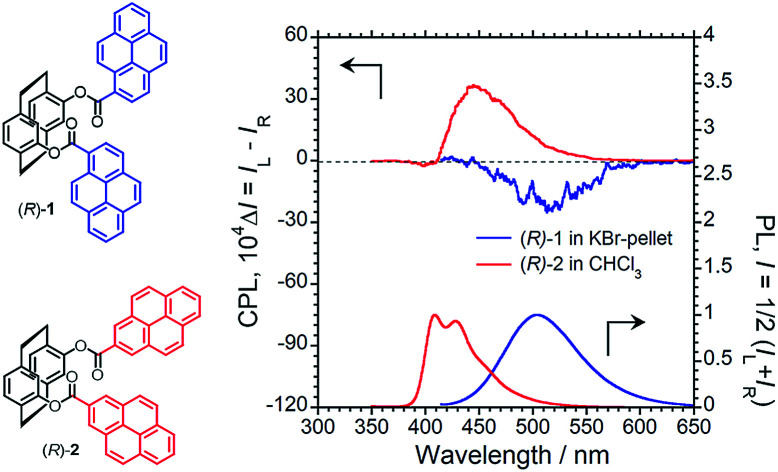

Right- and left-handed circularly polarised luminescence (CPL) with a high quantum yield (*Φ*_F_) and dissymmetry factor (*g* value) from enantiopairs of chiral luminescent materials has attracted considerable attention.^1^ Planar chiral [2,2]paracyclophane is a robust chiral skeleton that is useful for introducing π-conjugation into small and macromolecules. The Morisaki and Chujo groups have synthesised π-stacked chiral molecules based on [2,2]paracyclophane that exhibit CPL with high dissymmetry factors and high quantum yields.^2^ In contrast, Ema and co-workers reported intense excimer CPL from chiral quaternaphthyls containing four to eight pyrene units linked by ester groups.^3^

Recently, we reported that the non-classical CPL sign of chiral binaphthyl-pyrene organic luminophores with the same axial chirality could be controlled by changing the binding position of pyrene and selecting a specific linker between the chiral binaphthyl and fluorescent pyrene units or by introducing a binaphthyl unit with the opposite chirality.^4^ These chiral pyrene luminophores exhibited intramolecular excimer CPL in both solution and the solid state.

The aim of this work was to develop a novel CPL control system for switching between intra- and intermolecular excimer CPL in planar chiral paracyclophane-pyrene luminophores. For this purpose, we prepared two types of planar chiral paracyclophane-pyrene luminophores ((*R*)/(*S*)-1 and (*R*)/(*S*)-2) with different binding positions of the fluorescent pyrene units (Scheme 1). In 1 with a 1-pyrene unit, no CPL was observed in dilute CHCl_3_ solution, but green intermolecular excimer CPL at 510 nm occurred in the KBr-pellet. Constractingly, 2 with a 2-pyrene unit exhibited strong light blue intramolecular excimer CPL at 452 nm in dilute CHCl_3_ solution but no CPL in the KBr-pellet.

**Scheme 1:**
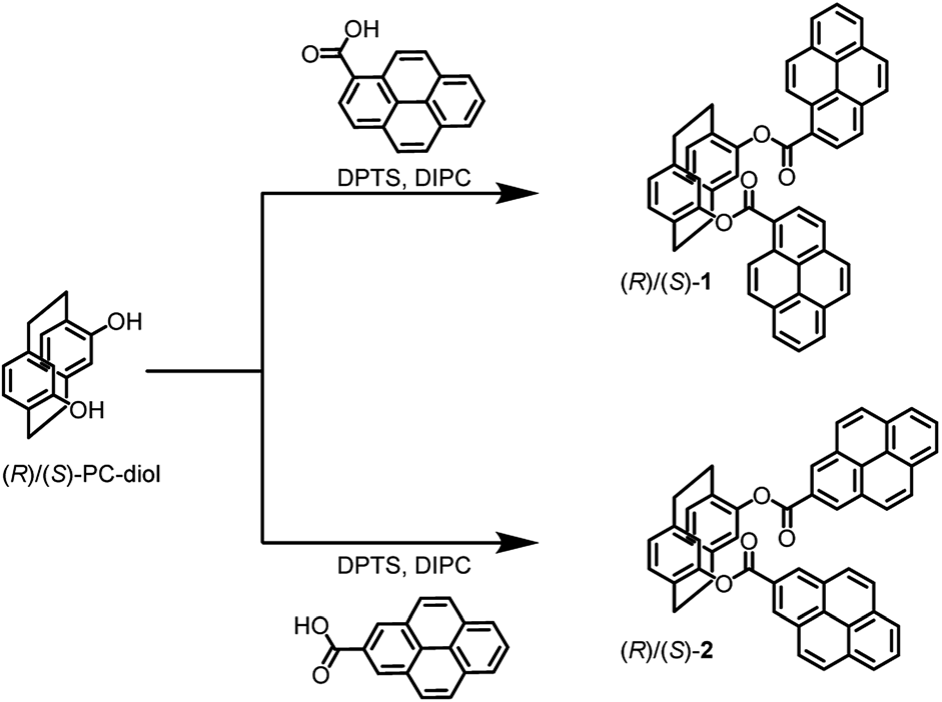
Synthesis of planar chiral paracyclophane-pyrene luminophores with different binding positions of pyrene fluorophores [(*R*)/(*S*)-1 and (*R*)/(*S*)-2]. DPTS: 4-(*N*,*N*-dimethylamino) pyridium-4-toluene sulfonate, DIPC: diisopropylcarbodiimide.

(*R*)/(*S*)-1 and (*R*)/(*S*)-2 were prepared using (*R*)/(*S*)-paracyclophanediol and 1-pyrenecarboxylic acid or 2-pyrenecarboxylic acid, respectively (Scheme 1).

We recorded the unpolarised photoluminescence (PL) and CPL properties of (*R*)/(*S*)-1 and (*R*)/(*S*)-2 in CHCl_3_ solution. As shown in Fig. 1(a), 1 with fluorescent 1-pyrene units does not exhibit a clear CPL signal in dilute CHCl_3_ solution (1.0 × 10^−4^ M), although monomer PL is observed with a maximum emission wavelength (*λ*_em_) of 393 nm.

**Fig. 1:**
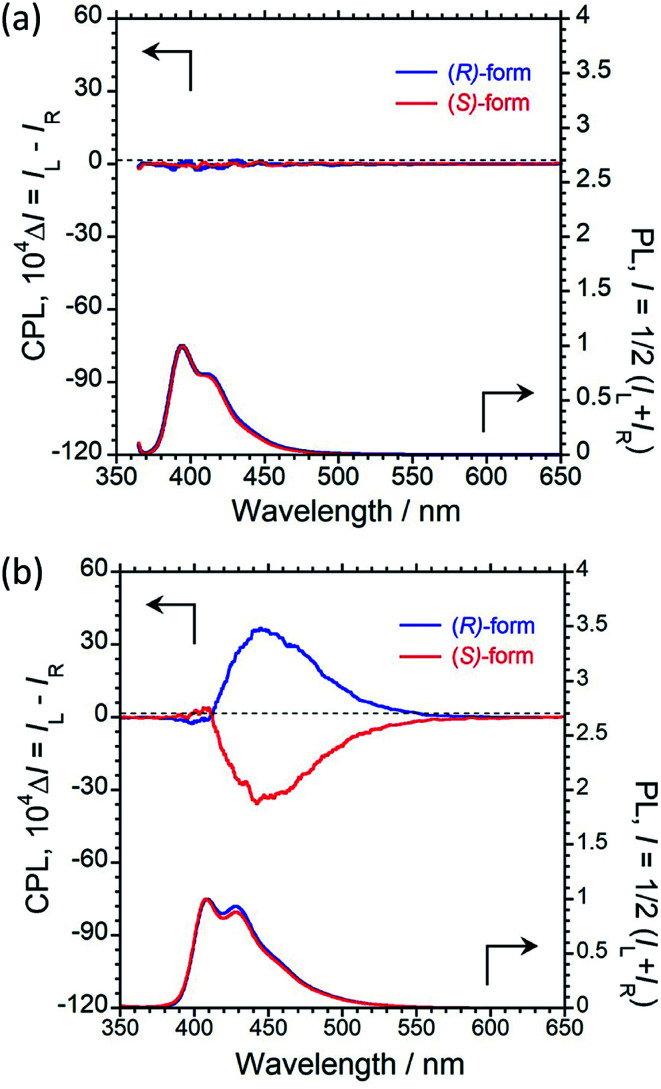
CPL (upper) and PL (lower) spectra of (a) (*R*)-1 (blue) and (*S*)-1 (red), and (b) (*R*)-2 (blue) and (*S*)-2 (red) in CHCl_3_ solution (1.0 × 10^−4^ M, *λ*_ex_ = 340 nm for 1 and 322 nm for 2, path length = 1 mm, 25 °C).

Interestingly, paracyclophane-pyrene luminophore 2 with fluorescent 2-pyrene units emitted clear CPL at a maximum CPL wavelength (*λ*_CPL_) of 452 nm, as shown in Fig. 1(b). This strong signal corresponded to excimer CPL derived from intramolecular π–π stacking of pyrenes, as the CPL band was similar in dilute and concentrated CHCl_3_ solutions (1.0 × 10^−5^ and 1.0 × 10^−3^ M) (Fig. S10 and S12†). The CPL capability was evaluated quantitatively using the equation *g*_CPL_ = Δ*I*/*I* = (*I*_L_ − *I*_R_)/[(*I*_L_ + *I*_R_)/2], where *I*_L_ and *I*_R_ are the output signal intensities for left- and right-handed circularly polarised light, respectively, under unpolarised photoexcitation conditions. The |*g*_CPL_| value at *λ*_CPL_ = 452 nm is 6.0 × 10^−3^ with an absolute PL quantum yield (*Φ*_F_) of 0.17%. The PL decay of (*R*)-2 in CHCl_3_ solution at 460 nm (Fig. S18†) consists of three dominant components (*τ*_1_ = 6.99 µs (20.4%), *τ*_2_ = 15.3 µs (61.8%), and *τ*_3_ = 1.02 µs (17.8%)). This finding indicates that at least three emissive species are responsible for the CPL at the π → π* transition of pyrene. These results suggest that simply changing the bonding position of the fluorescent pyrene units allowed switching of the CPL properties in the solution state.

We next recorded the circular dichroism (CD) and UV-vis absorption spectra to study the ground-state chirality of 1 and 2 in CHCl_3_ solution (1.0 × 10^−4^ M). Both (*R*)/(*S*)-1 and (*R*)/(*S*)-2 exhibited obvious mirror-image first Cotton effects at 390 and 339 nm, respectively, as shown in Fig. 2. The CD intensity originating from the ground-state chirality is known as the Kuhn's anisotropy factor and is theoretically defined as the dissymmetric factor: *g*_CD_ = (Δ*ε*_L_ − *ε*_R_) = 2(*ε*_L_ − *ε*_R_)/(*ε*_L_ + *ε*_R_). The |*g*_CD_| value for the first Cotton CD band is 0.58 × 10^−3^ at 390 nm for 1 (Fig. 2(a)) and 2.5 × 10^−3^ at 339 nm for 2 (Fig. 2(b)). This difference shows that the pyrene units in 1 and 2 are in different chiral environments in the solution state.

**Fig. 2:**
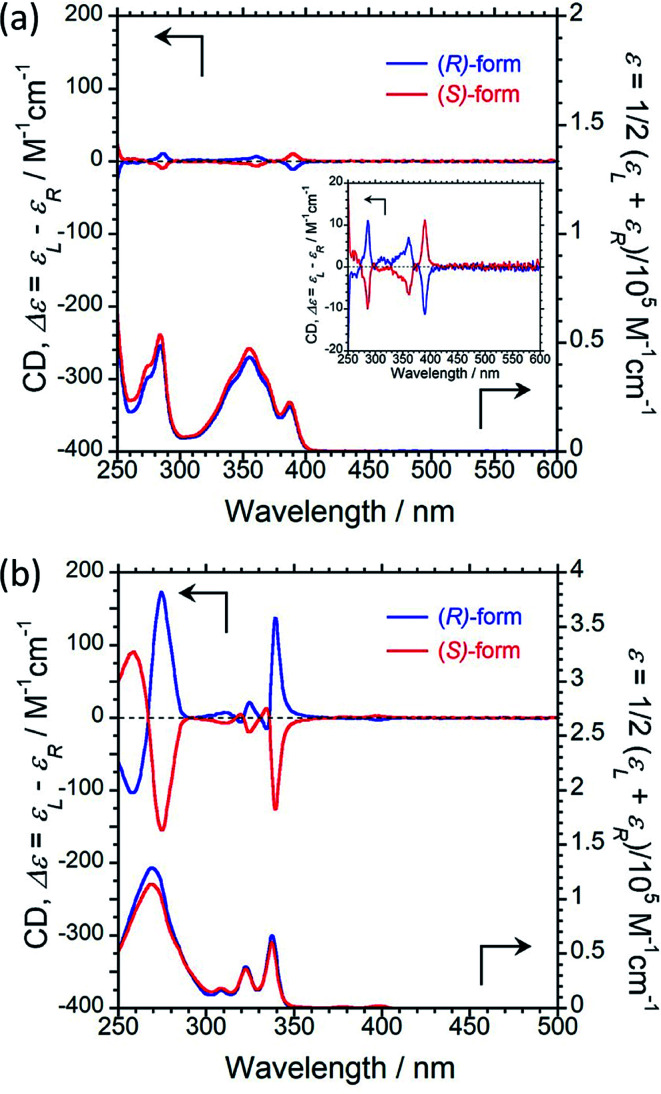
CD (upper) and UV-vis absorption (lower) spectra of (a) (*R*)-1 (blue) and (*S*)-1 (red), and (b) (*R*)-2 (blue) and (*S*)-2 (red) in CHCl_3_ solution (1.0 × 10^−4^ M, path length = 1 mm, 25 °C).

To investigate the HOMOs and LUMOs of the luminophores, we calculated the optimised structures using density functional theory (DFT) at the B3LYP/6-31G (d,p) level in the Gaussian 09 program.^5^ The optimised structures and the HOMOs/LUMOs of (*R*)-1 and (*R*)-2 are shown in Fig. 3. In 1, the HOMO is located on both the pyrene and paracyclophane units, and three vibronic UV bands (0–0′, 0–1′, and 0–2′) were calculated at 376, 373, and 371 nm by using time-dependent density functional theory (TD-DFT) at the B3LYP/6-31G (d,p) level in the Gaussian 09 program,^5^ respectively. On the contrast, the HOMO and LUMO in 2 are mainly located on the pyrene units, and three vibronic UV bands (0–0′, 0–1′, 0–2′) were calculated at 344, 328, and 314 nm, by using TD-DFT as same as 1, respectively. These values are similar to the experimental results, which suggests that the maximum absorption wavelength results from exciton coupling of the HOMO–LUMO π–π* electronic transition of two pyrene units.

**Fig. 3:**
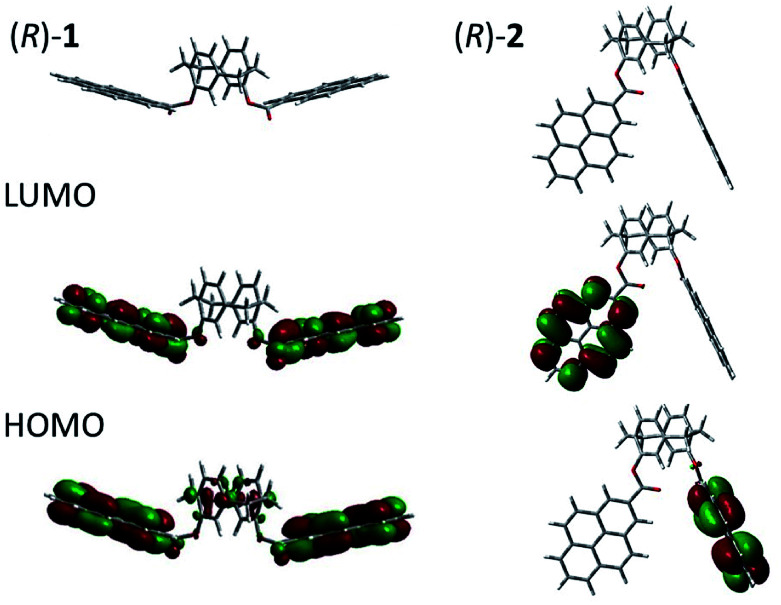
DFT-optimised structures and HOMOs/LUMOs of (*R*)-1 and (*R*)-2 at the B3LYP/6-31G (d,p) level of theory.

Subsequently, we studied the CPL behaviour of both (*R*)/(*S*)-1 and (*R*)/(*S*)-2 in the solid state (Fig. 4). Surprisingly, paracyclophane-pyrene luminophore 1 with fluorescent 1-pyrene units emitted strong green CPL at 510 nm in the KBr-pellet, even though no CPL was observed in dilute CHCl_3_ solution. The weak CPL spectrum observed in concentrated CHCl_3_ solution (1.0 × 10^−3^ M) showed the same emission bands as in the KBr-pellet (Fig. S7†). This result indicates that the green CPL is excimer emission derived from the intermolecular π–π stacking of pyrenes in the solid state. The |*g*_CPL_| value at the maximum CPL wavelength (513 nm) was 3.9 × 10^−3^ and the *Φ*_F_ value was 0.03. The PL decay of (*R*)-1 in the solid state at 510 nm consisted of three components (*τ*_1_ = 3.84 µs (11.7%), *τ*_2_ = 19.0 µs (80.5%), and *τ*_3_ = 0.3 µs (7.83%)) (Fig. S17†). Thus, at least three emissive species are responsible for the CPL properties at the π → π* transition of pyrene.

**Fig. 4:**
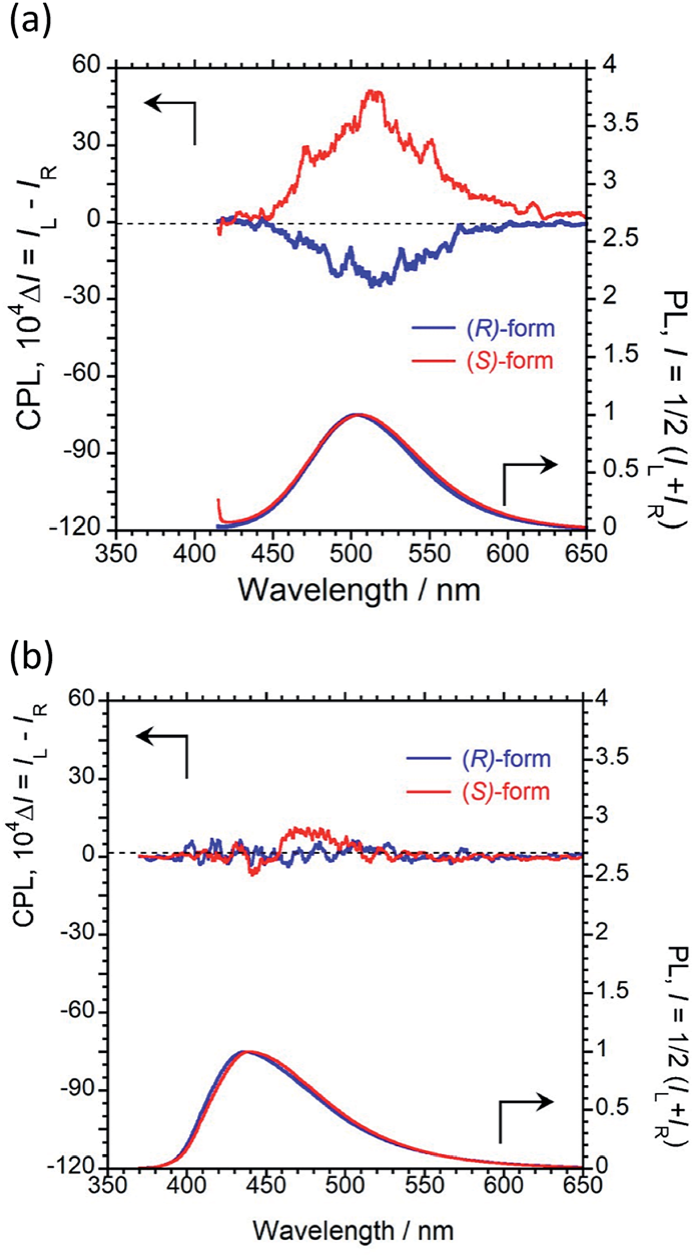
CPL (upper) and PL (lower) spectra of (a) (*R*)-1 (blue) and (*S*)-1 (red), and (b) (*R*)-2 (blue) and (*S*)-2 in the KBr-pellet (*λ*_ex_ = 388 nm for 1 and 344 nm for 2, 25 °C).

On the contrast to 1, paracyclophane-pyrene luminophore 2 with fluorescent 2-pyrene units exhibited no clear excimer CPL in the KBr-pellet, although monomer PL was observed at 440 nm.

To study the ground-state chirality in the KBr-pellet, the CD and UV-vis absorption spectra of (*R*)/(*S*)-1 and (*R*)/(*S*)-2 were recorded, as shown in Fig. 5. In the KBr-pellet, opposite CD signals were observed for (*R*)/(*S*)-1 and (*R*)/(*S*)-2. As in solution, a negative Cotton effect was observed for (*R*)-1 and a positive Cotton effect was observed for (*R*)-2 despite the two luminophores having the same chiral skeleton. The |*g*_CD_| values of the first Cotton band were 0.18 × 10^−3^ at 390 nm for 1 and 1.2 × 10^−3^ at 339 nm for 2. This result shows that the ground-state chirality of the pyrene units in 1 and 2 are different in the KBr-pellet. However, for either 1 or 2, the ground-state chiral environments of the pyrene units are similar in both CHCl_3_ solution and the KBr-pellet.

**Fig. 5:**
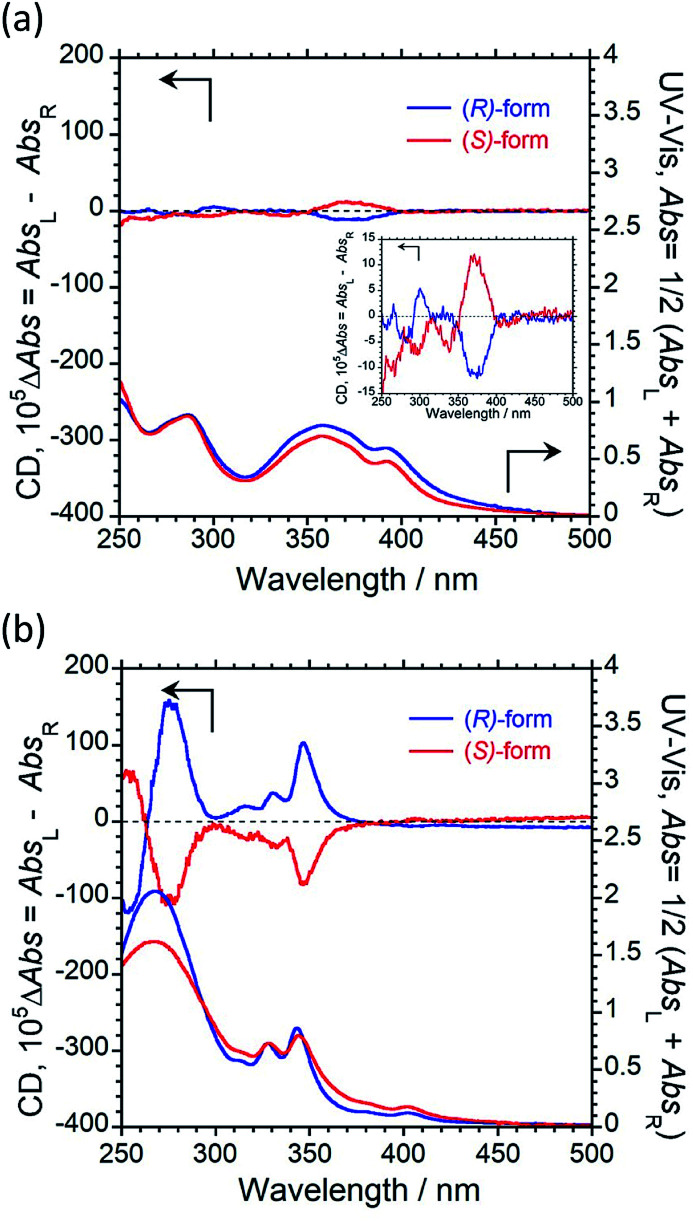
CD (upper) and UV-vis absorption (lower) spectra of (a) (*R*)-1 (blue) and (*S*)-1 (red), and (b) (*R*)-2 (blue) and (*S*)-2 in the KBr-pellet at 25 °C.

Based on these results, it is thought that in 1, which bears fluorescent 1-pyrene units, the two pyrene units cannot form an intramolecular excimer chiral configuration in the photoexcited state in solution; however, in the photoexcited state in the solid state, two pyrene units on different molecules can form an intermolecular excimer chiral configuration. Constratingly, in 2, which bears fluorescent 2-pyrene units, the two pyrene units can form an intramolecular excimer chiral configuration in the photoexcited state in solution, but two pyrene units on different molecules cannot form an intermolecular excimer chiral configuration in the photoexcited state in the solid state. This switching of the CPL characteristics is considered to arise from the difference in the orientation of the two pyrene units in 1 and 2.

In summary, we designed two types of planar chiral paracyclophane-pyrene luminophores. The CPL properties of these luminophores depended on the binding position of the fluorescent pyrene units. Compound 1 with 1-pyrene units showed green intermolecular excimer CPL in the KBr-pellet despite exhibiting no CPL in dilute CHCl_3_ solution. In contrast, 2 with 2-pyrene units showed light blue intramolecular excimer CPL in CHCl_3_ solution but no CPL was observed in the KBr-pellet. This is a first report of clear switching of intra- or intermolecular excimer CPL by changing the binding position of fluorescent pyrene units.

## Conflicts of interest

There are no conflicts to declare.

## Supplementary Material

RA-010-D0RA01552K-s001
