# Supplementary material for: Inter- and intramolecular excimer circularly polarised luminescence of planar chiral paracyclophane-pyrene luminophores
Source: RSC Adv. 2020 Mar 20;10(19):11335–8. doi: 10.1039/d0ra01552k (PMC9050457; doi:10.1039/d0ra01552k)
Supplement: RA-010-D0RA01552K-s001 [file RA-010-D0RA01552K-s001.pdf]

Electronic Supplementary Material (ESI) for RSC Advances.
This journal is © The Royal Society of Chemistry 2020

Electronic Supporting Information (ESI) for

Inter- and intramolecular excimer circularly polarised luminescence of planar chiral paracyclophane-pyrene luminophores

Nobuyuki Hara,[a] Motohiro Shizuma,[b] Takunori Harada[c] and Yoshitane Imai*[a]

[a] Department of Applied Chemistry, Faculty of Science and Engineering, Kindai University, 3-4-1 Kowakae, Higashi-Osaka, Osaka 577-8502, Japan. E-mail: y-imai@apch.kindai.ac.jp
[b] Department of Biochemistry, Osaka Research Institute of Industrial Science and Technology, 1-6-50 Morinomiya, Joto-ku, Osaka 536-8553, Japan.
[c] Department of Integrated Science and Technology, Faculty of Science and Technology, Oita University, Dannoharu, 700, Oita City 870-1192, Japan

Table of Contents

1. **Materials** **S3**
2. **Experimental section** **S3**
3. **Synthesis of compounds** **S5**
4. **CPL and PL spectra of (*R*)- and (*S*)-1 in $CHCl_3$ solution** **S10**
5. **CPL and PL spectra of (*R*)- and (*S*)-2 in $CHCl_3$ solution** **S11**
6. **CD and UV-vis absorption spectra of (*R*)- and (*S*)-1 in $CHCl_3$ solution** **S13**
7. **CD and UV-vis absorption spectra of (*R*)- and (*S*)-2 in $CHCl_3$ solution** **S14**
8. **Lifetime decay curves of (*R*)-1 and (*R*)-2** **S15**

1. Materials

(*R*)- and (*S*)-4,12-Dihydroxy-[2,2]-paracyclophane and 1-pyrenecarboxylic acid were purchased from Daicel Corporation and Tokyo Chemical Industry (TCI), respectively. 2-Pyrenecarboxylic acid and 4-(N,N-dimethylamino)pyridinium-4-toluenesulfonate (DPTS) were prepared by previously reported methods.[S1] KBr for the formation of solid pellets was purchased form Wako Pure Chemical Corp. and $CHCl_3$ for solution state measurements was purchased from FUJIFILM Wako Pure Chemical Corp.

2. Experimental

2.1. Absolute PL quantum yields

The absolute PL quantum yields were measured using an Absolute PL Quantum Yield Measurement System (C9920-02, Hamamatsu Photonics) in air at room temperature. The excitation wavelengths were 340 nm for **1** and 322 nm for **2** in $CHCl_3$ solution (path length 10 mm) and in 388 nm for **1** and 322 nm for **2** in the KBr-pellet.

2.2. PL and CPL spectra

The PL and CPL spectra were recorded using a JASCO CPL-300 spectrofluoropolarimeter at room temperature. A scattering angle of 0° was used for the non-polarised monochromatic incident light with an excitation bandwidth of 10 nm and an emission bandwidth of 10 nm. The scanning speed was 50 nm min^{-1} and the PMT time constant was 8 s. The excitation wavelengths were 340 nm for **1** and 322 nm for **2** in $CHCl_3$ solution (path length 1 mm for 1.0×10^{-3} M and 10^{-4} M, 10 mm for 1.0×10^{-5} M) and 388 nm for **1** and 322 nm for **2** in the KBr-pellet. The solid-state samples were prepared according to the standard procedure for obtaining glassy KBr matrices. [S2]

2.3. CD and UV-vis absorption spectra

The CD and UV-vis absorption spectra in $CHCl_3$ solution (path length 1 mm) were recorded using a JASCO J-820 spectropolarimeter at room temperature. The CD and UV-vis absorption spectra in the KBr-pellet were recorded using a solid-state dedicated chiroptical spectrophotometer (J-1500, JASCO) at room temperature. The solid-state samples were prepared according to the standard procedure for obtaining glassy KBr matrices.

2.4. NMR spectra

^{1}H- and ^{13}C-NMR spectra were recorded using a JEOL ECA 600 spectrometer. Tetramethyl silane (Me_4Si) was used as an internal reference.

2.5. Mass spectra

Mass spectra were recorded using an AXIMA Confidence mass spectrometer (Shimazdu Co.) under the following conditions: ionisation method, laser desorption ionisation method; matrix, none; mass range, *m/z* 1–3000; mode, reflection (negative); laser power, 65–95. Calibration was performed using C_{60} (*m/z* 720.0005 for $[M]^-$), angiotensin II (*m/z* 1044.5272 for $[M - H]^-$), and CHCA (*m/z* 189.0431 for $[M - H]^-$) as external standards.

2.6. PL decay dynamics

The PL decays were recorded using a Fluoromax-4 spectrofluorometer (Horiba Co.) at 510 nm for (*R*)-**1** in the solid state and 450 nm for (*R*)-**2** in $CHCl_3$ solution under the following conditions: measurement range, 800 ns; excitation wavelength, 295 nm (nano LED); peak pre-set, 10000 counts; repetition rate, 1 MHz; sync delay, 30 ns.

References

S1. P. Rajakumar and K. Visalakshi. *Synth. Commun.* 2013, **43**, 2226.

S2. R. Kuroda and Y. Saito. *Bull. Chem. Soc. Jpn.* 1976, **49**, 433.

3. Synthesis of compounds ((*R*)/(*S*)-1 and (*R*)/(*S*)-2)

3.1. Synthesis of (*R*)/(*S*)-1

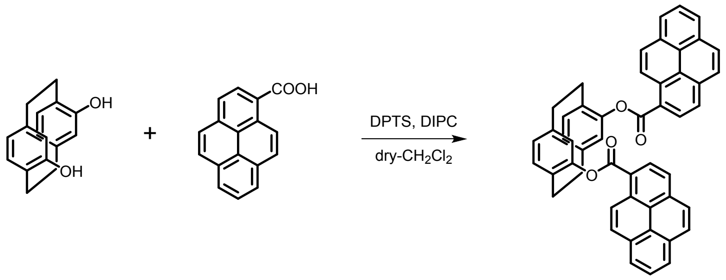

(*R*)-4,12-Dihydroxy-[2,2]-paracyclophane (10 mg, 0.042 mmol) and 1-pyrenecarboxylic acid (21.5 mg, 0.087 mmol) were dissolved in dry CH_2Cl_2 under N_2. DPTS (12.4 mg, 0.042 mmol) was added to the solution mixture at r.t. After 15 min, diisopropylcarbodiimide (DIPC) (0.1 mL, 0.6 mmol) was added dropwise, and the mixture was stirred for 17 h. The mixture was extracted with dichloromethane and washed with water. The combined organic layer was dried over $MgSO_4$, concentrated, and purified by silica gel column chromatography using dichloromethane/hexane (1:3, v/v) as the eluent to obtain (*R*)-**1** (12 mg, 44% yield) as a yellow solid. (*S*)-**1** was prepared in an identical manner from (*S*)- 4,12-~~dihydroxy~~Dihydroxy-[2,2]-paracyclophane to give the desired product in 37% yield.

^{1}H NMR (600 MHz, $CDCl_3$) δ = 9.39 (d, *J* = 9.6 Hz, 2H), 9.00 (d, *J* = 7.8 Hz, 2H), 8.24 (d, *J* = 7.8 Hz, 2H), 8.18–8.17 (m, 4H), 8.11 (d, *J* = 7.8 Hz, 2H), 8.08 (d, *J* = 9.6 Hz, 2H), 8.04 (d, *J* = 9.6 Hz, 2H), 8.02 (t, *J* = 7.8 Hz, 2H), 7.03 (s, 2H), 6.77 (d, *J* = 7.8 Hz, 2H), 6.62 (dd, *J* = 7.8 Hz, 1,8 Hz, 2H), 3.44–3.40 (m, 2H), 3.18 (t, *J* = 7.2 Hz, 4H), 2.92–2.87 (m, 2H); ^{13}C NMR (150 MHz, $CDCl_3$) δ = 165.8, 149.6, 142.1, 135.6, 134.9, 131.9, 131.3, 131.0, 130.8, 130.3, 130.0, 129.9, 128.5, 127.1, 126.6, 126.4, 124.9, 124.8, 124.7, 124.4, 124.1, 123.9, 34.1, 31.5, 29.7; HRMS, Found *m/z* 696.2297, Calcd. *m/z* 696.2306 for $[M]^-$ (M: $C_{50}H_{32}O_4$).

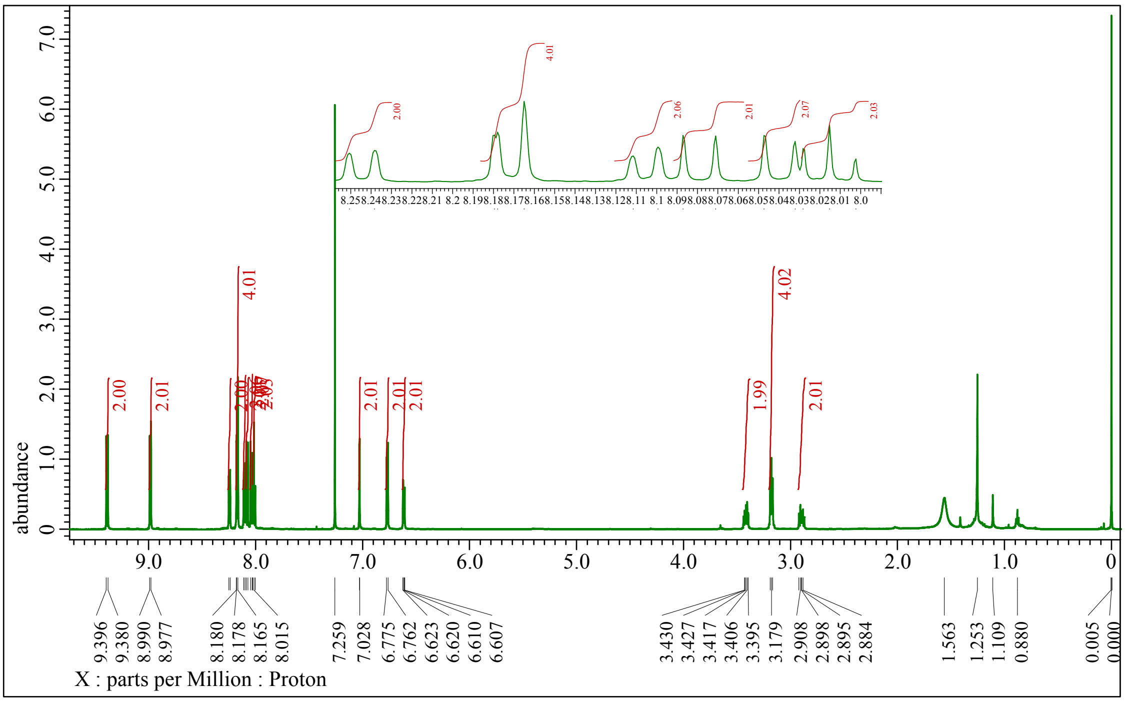

Fig. S1. ^{1}H-NMR spectrum of (*R*)-**1** (600 MHz, $CDCl_3$, 298 K, Me_4Si).

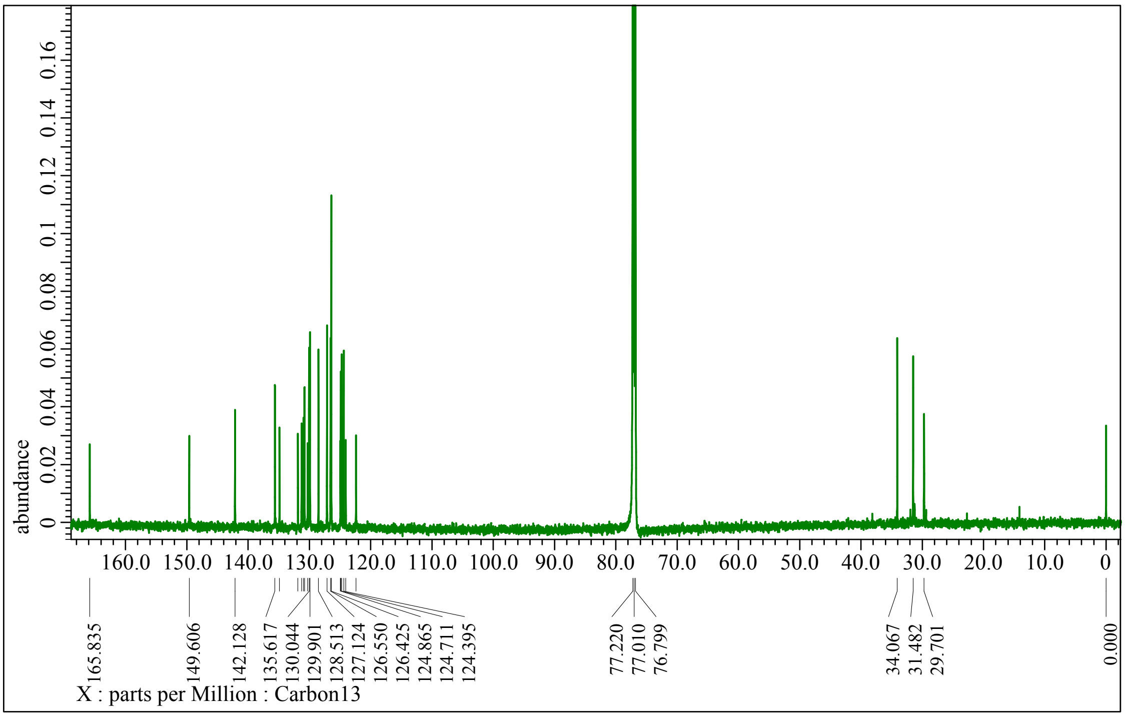

Fig. S2. ^{13}C-NMR spectrum of (*R*)-**1** (150 MHz, $CDCl_3$, 298 K, Me_4Si).

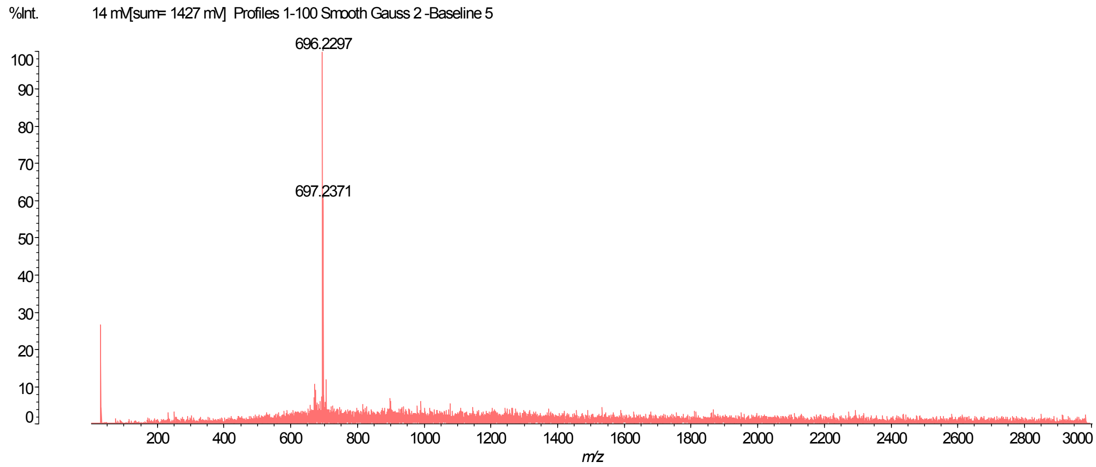

Fig. S3. Mass spectrum of (*R*)-**1**.

3.2. Synthesis of (*R*)/(*S*)-2

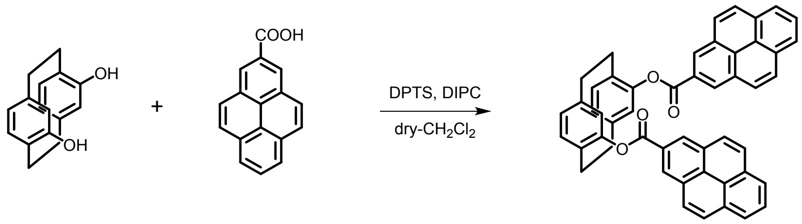

(*R*)-4,12-Dihydroxy-[2,2]-paracyclophane (10 mg, 0.042 mmol) and 2-pyrenecarboxylic acid (22.0 mg, 0.089 mmol) were dissolved in dry CH_2Cl_2 under N_2. Then DPTS (12.4 mg, 0.042 mmol) was added to the solution mixture at r.t. After 15 min, DIPC (0.1 mL, 0.6 mmol) was added dropwise, and the mixture was stirred for 17 h. The mixture was extracted with dichloromethane and washed with water. The combined organic layer was dried over $MgSO_4$, concentrated, and purified by silica gel column chromatography using dichloromethane/hexane (1:1, v/v) as the eluent to obtain (*R*)-**2** (9 mg, 31% yield) as a white solid. (*S*)-**2** was prepared in an identical manner from (*S*)-4,12-~~dihydroxy~~Dihydroxy-[2,2]-paracyclophane to give the desired product in 76% yield.

^{1}H NMR (600 MHz, $CDCl_3$) δ = 9.02 (s, 4H), 8.14 (d, *J* = 7.2 Hz, 4H), 8.04–7.97 (m, 10H), 7.10 (s, 2H), 6.77 (d, *J* = 8.4 Hz, 2H), 6.63 (dd, *J* = 7.2 Hz, 2H), 3.43–3.39 (m, 2H), 3.28–3.16 (m, 4H), 2.93–2.89 (m, 2H); ^{13}C NMR (150 MHz, $CDCl_3$) δ = 165.2, 149.7, 142.1, 135.4, 131.7, 131.2, 131.1, 131.0, 128.3, 127.6, 127.1, 126.6, 125.9, 125.5, 124.6, 124.1, 34.2, 31.3, 29.7; HRMS, Found *m/z* 696.2297, Calcd. *m/z* 696.2306 for $[M]^-$ (M: $C_{50}H_{32}O_4$).

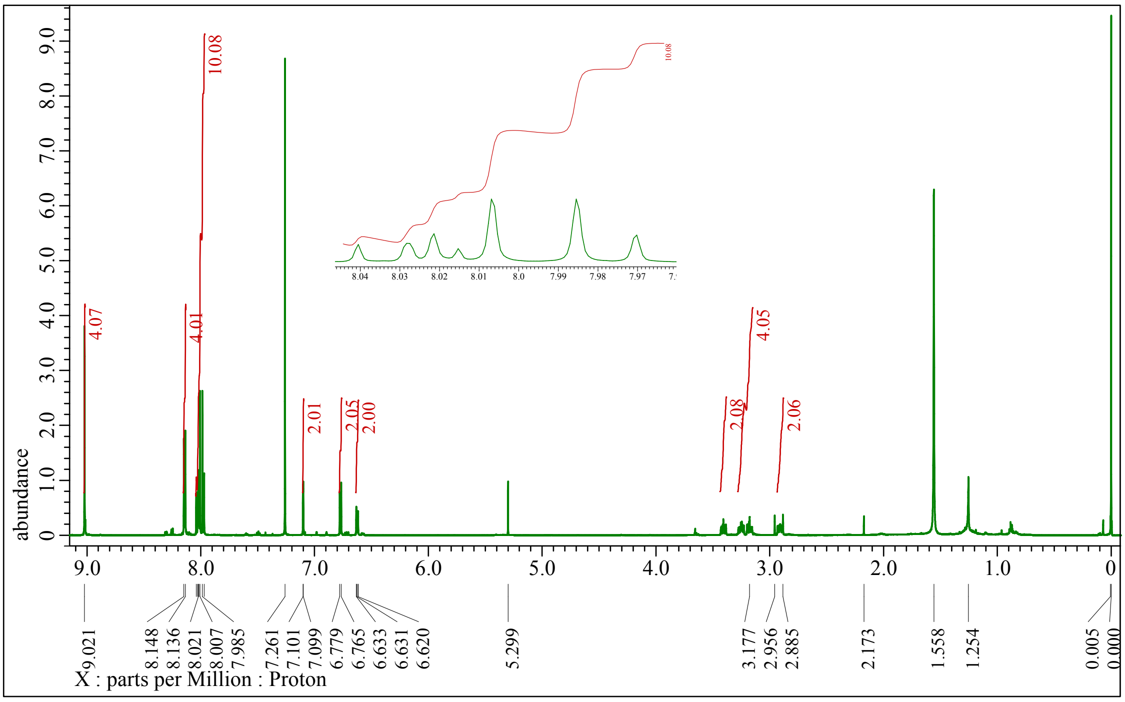

Fig. S4. ^{1}H-NMR spectrum of (*R*)-**2** (600 MHz, $CDCl_3$, 298 K, Me_4Si).

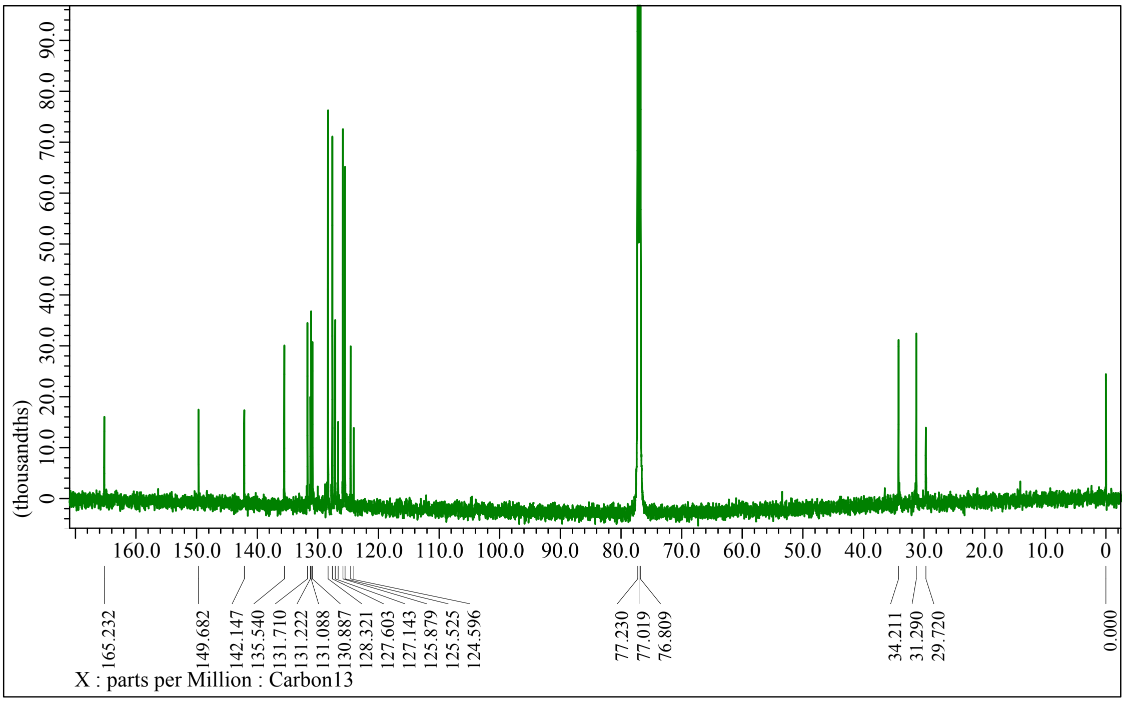

Fig. S5. ^{13}C-NMR spectrum of (*R*)-**2** (150 MHz, $CDCl_3$, 298 K, Me_4Si).

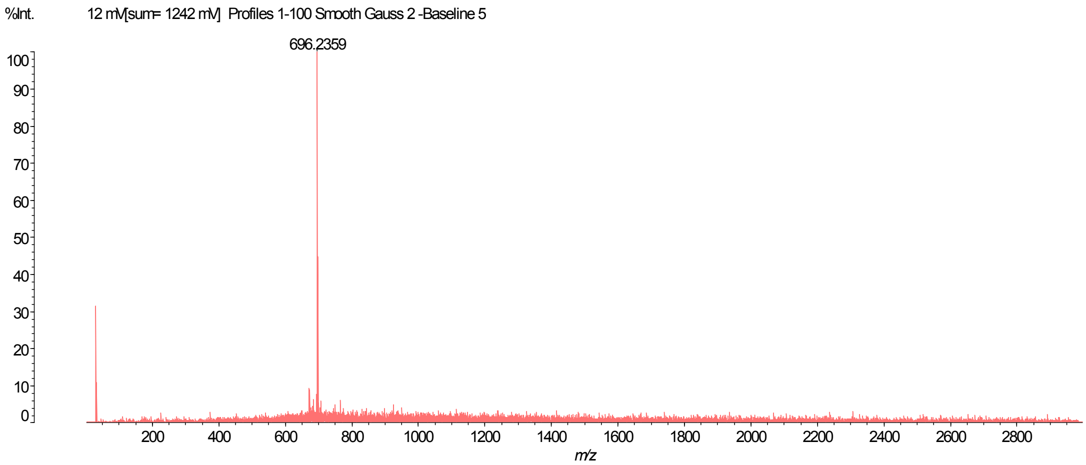

Fig. S6. Mass spectrum of (*R*)-**2**.

4. CPL and PL spectra of (*R*)/(*S*)-1 in $CHCl_3$ solution

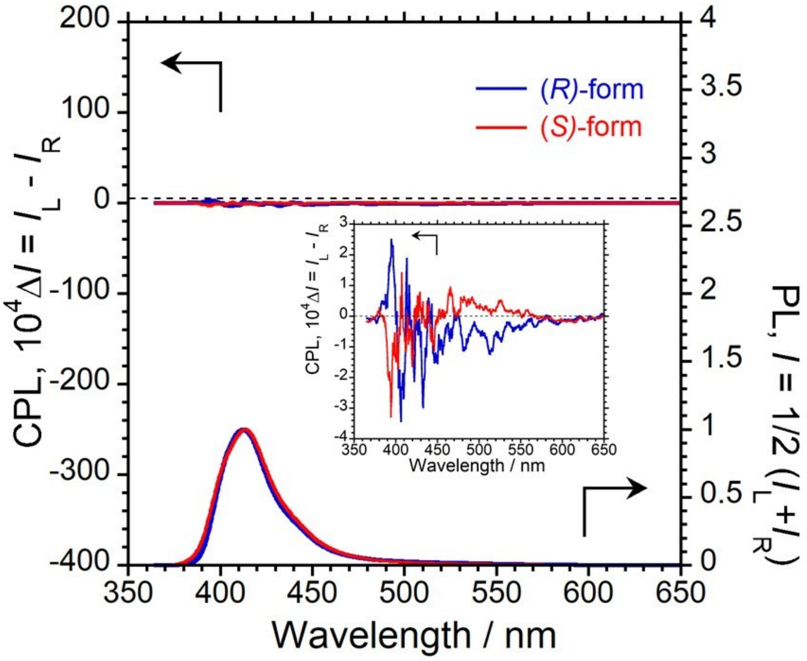

Fig. S7. CPL (upper) and PL (lower) spectra of **1** in $CHCl_3$ solution (1.0×10^{-3} M, λ_{ex} = 340 nm, path length = 1 mm, 25 °C).

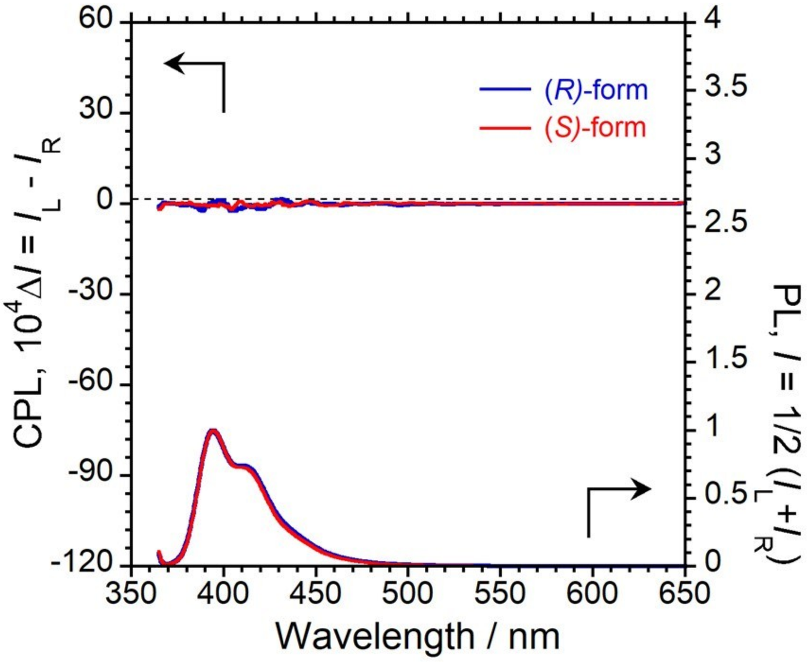

Fig. S8. CPL (upper) and PL (lower) spectra of **1** in $CHCl_3$ solution (1.0×10^{-4} M, λ_{ex} = 340 nm, path length = 1 mm, 25 °C).

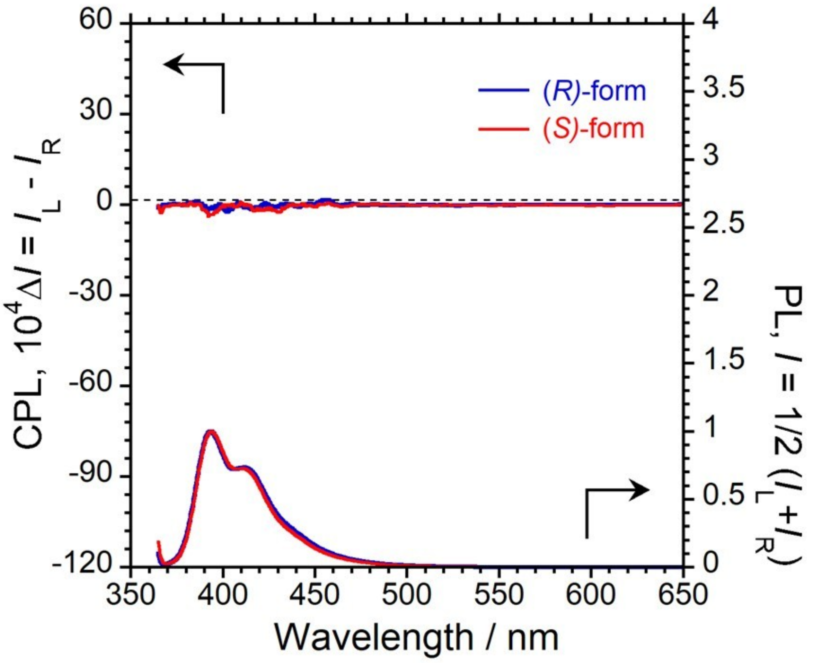

Fig. S9. CPL (upper) and PL (lower) spectra of **1** in $CHCl_3$ solution (1.0×10^{-5} M, λ_{ex} = 340 nm, path length = 10 mm, 25 °C).

5. CPL and PL spectra of (*R*)/(*S*)-2 in $CHCl_3$ solution state

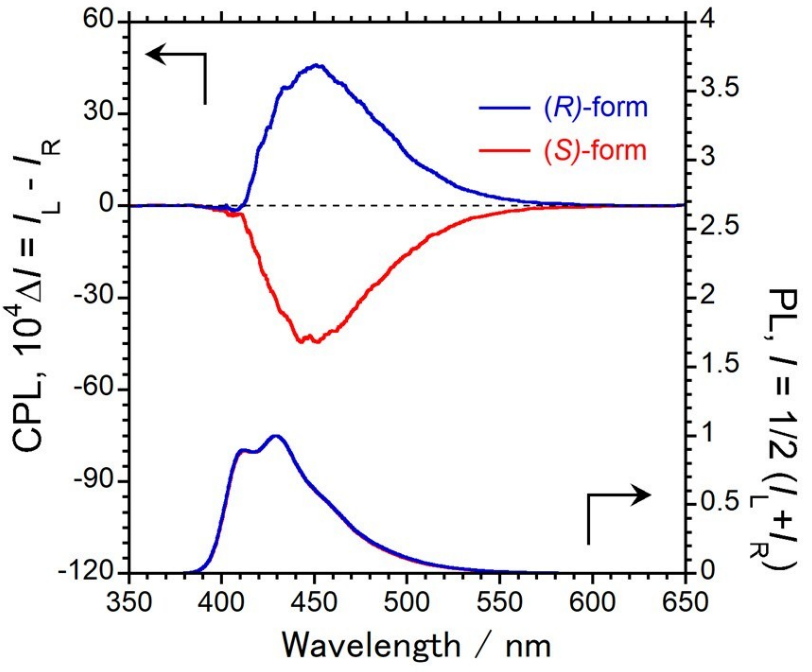

Fig. S10. CPL (upper) and PL (lower) spectra of **2** in $CHCl_3$ solution (1.0×10^{-3} M, λ_{ex} = 32244 nm, path length = 1 mm, 25 °C).

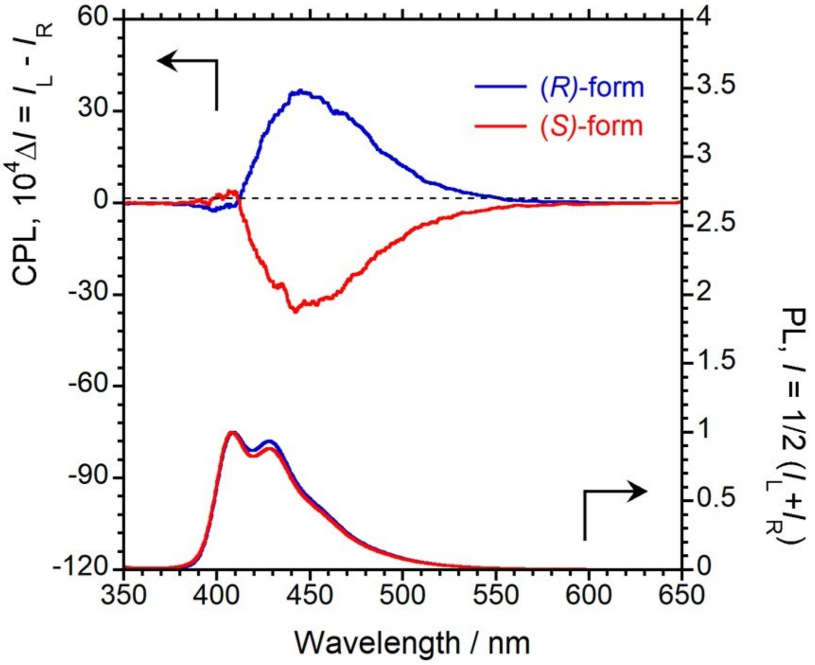

Fig. S11. CPL and PL spectra of **2** in $CHCl_3$ solution

(1.0 × 10^{-4} M, λ_{ex} = 322344 nm, path length = 1 mm, 25 °C).

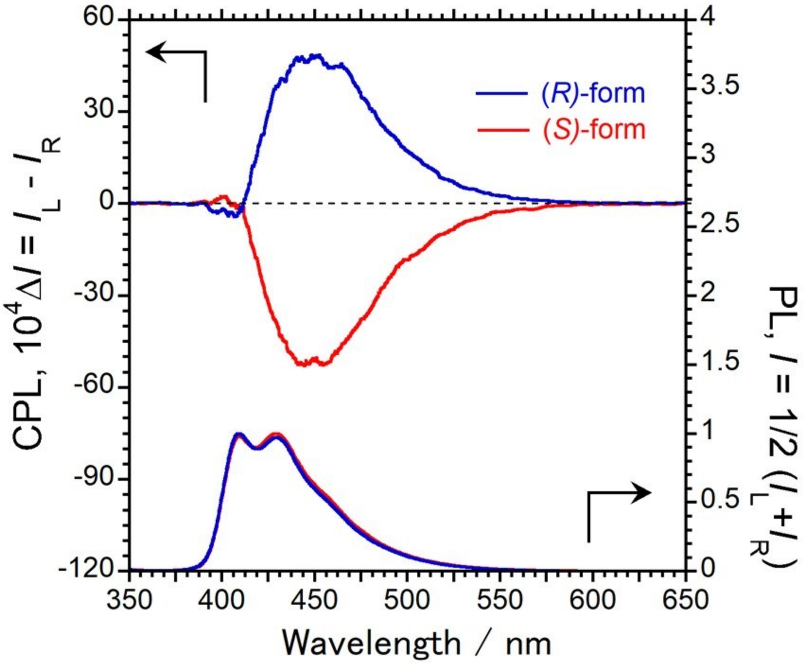

Fig. S12. CPL and PL spectra of **2** in $CHCl_3$ solution

(1.0 × 10^{-5} M, λ_{ex} = 322344 nm, path length = 10 mm, 25 °C).

6. CD and UV-vis absorption spectra of (*R*)/(*S*)-1 in $CHCl_3$ solution

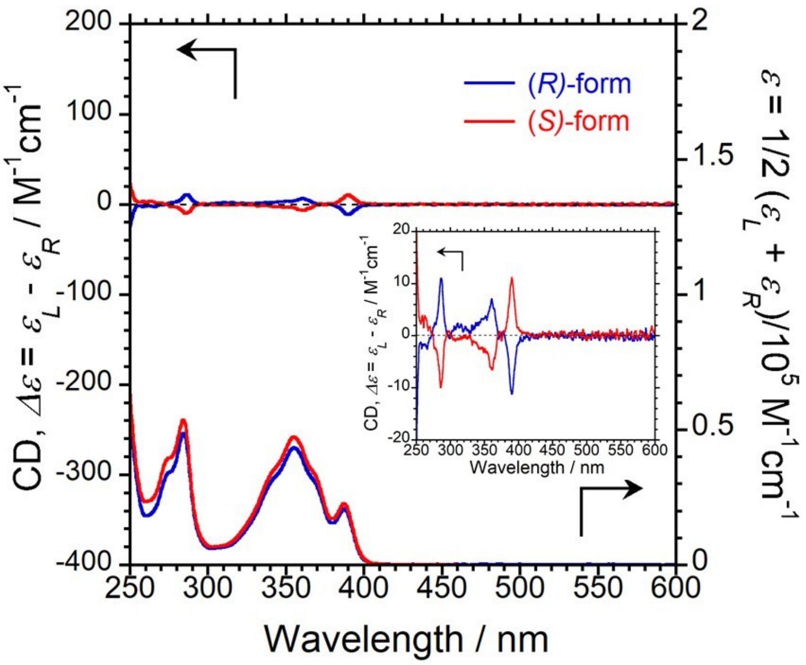

Fig. S13. CD (upper) and UV-vis absorption (lower) spectra of **1** in $CHCl_3$ solution (1.0×10^{-4} M, path length = 1 mm, 25 °C).

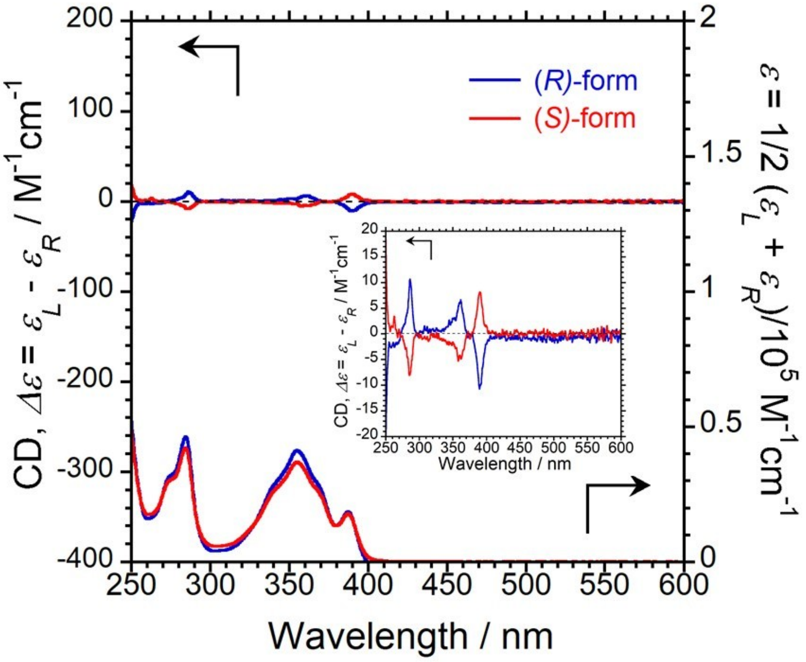

Fig. S14. CD (upper) and UV-vis absorption (lower) spectra of **1** in $CHCl_3$ solution (1.0×10^{-5} M, path length = 10 mm, 25 °C).

7. CD and UV-vis absorption spectra of (*R*)/(*S*)-2 in $CHCl_3$ solution

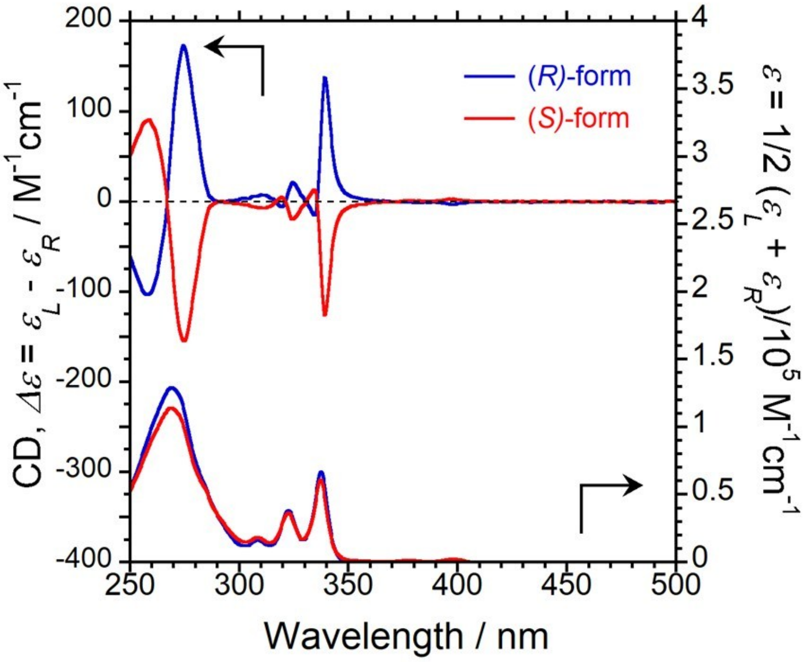

Fig. S15. CD (upper) and UV-vis absorption (lower) spectra of **2** in $CHCl_3$ solution (1.0×10^{-4} M, path length = 1 mm, 25 °C).

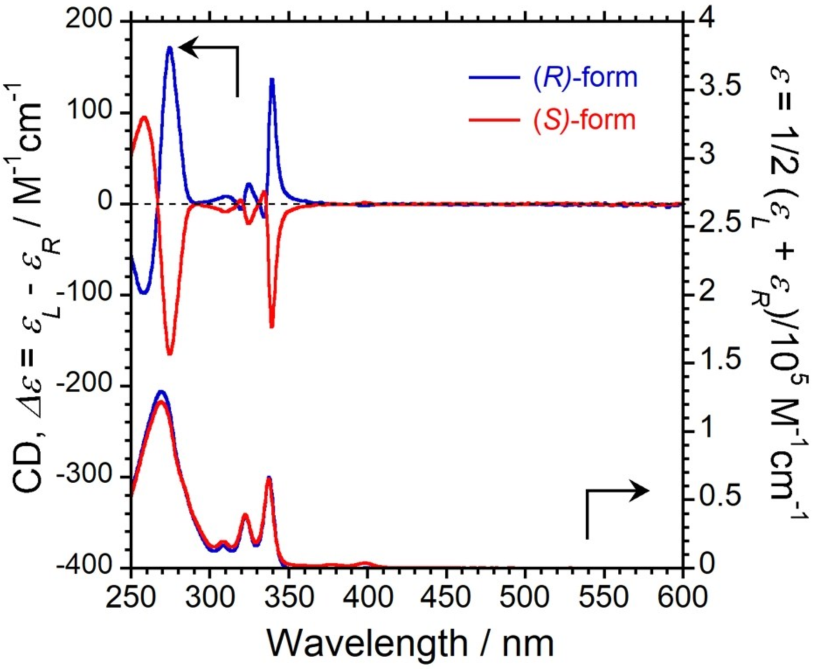

Fig. S16. CD (upper) and UV-vis absorption (lower) spectra of **2** in $CHCl_3$ solution (1.0×10^{-5} M, path length = 10 mm, 25 °C).

8. Lifetime decay curves of (*R*)-1 and (*R*)-2

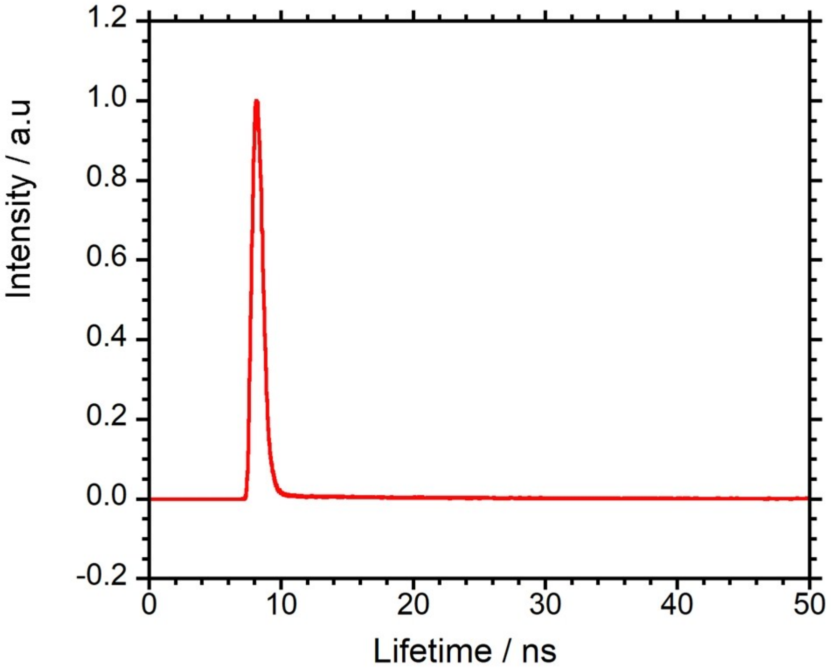

Fig. S17. Lifetime decay curve of (*R*)-**1** in the KBr-pellet.

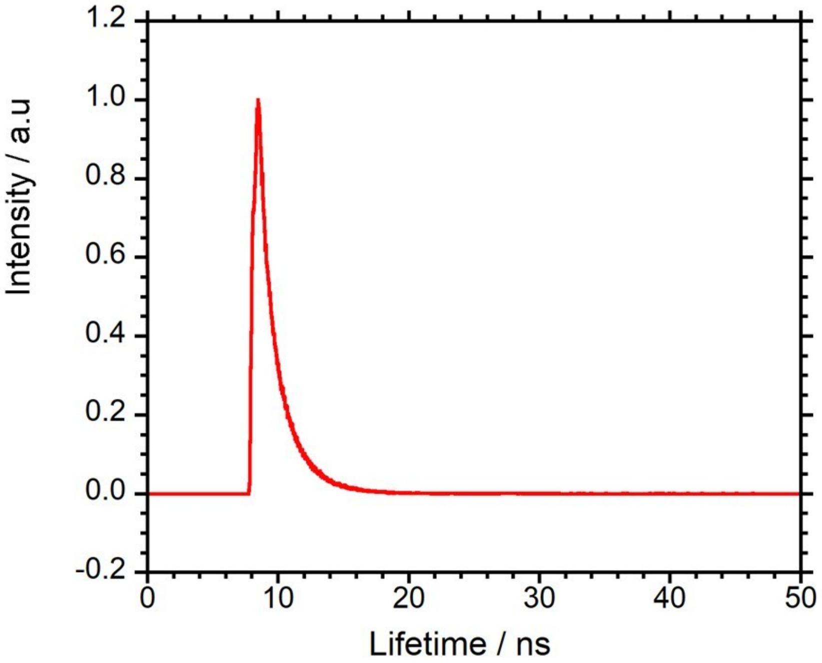

Fig. S18. Lifetime decay curve of (*R*)-**2** in $CHCl_3$ solution.
